# Supplementary material for: Risk and outcomes of healthcare-associated infections in three hospitals in Bobo Dioulasso, Burkina Faso, 2022: A longitudinal study
Source: PLoS One. 2025 Feb 14;20(2):e0307346. doi: 10.1371/journal.pone.0307346 (PMC11828398; doi:10.1371/journal.pone.0307346)
Supplement: S1 Appendice — (DOCX) [file pone.0307346.s001.docx]

**Appendice1: Microbial identification and antimicrobial susceptibility testing:**

***Determination of BMR susceptibility phenotype***

Samples taken for diagnostic purposes will undergo the following bacteriological tests: fresh state, Gram stain, culture on solid agar. Microbial identification from positive cultures was achieved by culture morphology and biochemical identification tests. For the identification of Enterobacteriaceae and non-fermenting Gram-negative bacilli, API 20 E and API 20 NE strips (BioMérieux) were used. For Gram-positive cocci, selective media and conventional biochemical tests were used for identification. Culture results were evaluated by a senior biologist to distinguish between pathogens and contaminants.

***Conservation of strains***

While waiting for genotypic determination of resistance, bacterial strains will be stored in aliquots of heart-brain broth with 10% glycerol added at -80°C.
