# Supplementary material for: Risk and outcomes of healthcare-associated infections in three hospitals in Bobo Dioulasso, Burkina Faso, 2022: A longitudinal study
Source: PLoS One. 2025 Feb 14;20(2):e0307346. doi: 10.1371/journal.pone.0307346 (PMC11828398; doi:10.1371/journal.pone.0307346)
Supplement: S2 Table — (DOCX) [file pone.0307346.s002.docx]

**S2_Table: Definitions of HAI Cases Monitored**

***Urinary Tract Infections***

Patients who have had a urinary catheter inserted within the previous 7 days and who have at least two of the following symptoms without other known cause: Fever (> 38°C) without other infectious site; Urge to urinate; Dysuria; Pollakiuria; Suprapubic tenderness. At least one of the following Pyuria >10 leucocytes/ml; observation of microorganisms on Gram stain, non-centrifuged urine; appropriate treatment ongoing. AND positive uroculture (≥105 microorganisms/ml) with no more than two microbial species isolated.

***Sepsis/bacteremia***

Fever >38°C with at least one of the following: hypotension (systolic pressure <90 mm Hg) or oliguria (<0.5 ml/kg/h), chills, tachycardia, polypnea, with at least one positive blood culture in the absence of another cause.

***Surgical Site Infection***

Any purulent discharge, surgical abscess or extensive cellulitis at the surgical site within 30 days of surgery or three months if a prosthesis is in place. Or any sign of infection (fever above 38°C, pain, tenderness, redness, heat, etc.) associated with the intentional opening of the superficial part of the incision by the surgeon.

**Newborn infection**

Newborn with at least 2 of the following within 48 hours of birth Temperature > 38°C or < 36.5°C or unstable temperature; tachycardia or bradycardia; apnea; skin redness time greater than 2 seconds; other signs such as lethargy. Excluding a context of premature rupture of membranes, maternal chorioamnionitis, disease present or incubating in the mother. With blood or CSF culture positive for a recognized pathogen.
