# Supplementary material for: Risk and outcomes of healthcare-associated infections in three hospitals in Bobo Dioulasso, Burkina Faso, 2022: A longitudinal study
Source: PLoS One. 2025 Feb 14;20(2):e0307346. doi: 10.1371/journal.pone.0307346 (PMC11828398; doi:10.1371/journal.pone.0307346)
Supplement: S4 Table — (DOCX) [file pone.0307346.s004.docx]

**S4_Table :** Distribution of adult and pediatric patients according to HAI data, multicenter longitudinal survey, Bobo-Dioulasso 2022

|  | **All patients**  **n (%)** | **Adults**  **n (%)** | **children n(%)** | **p-value** |
| --- | --- | --- | --- | --- |
| **All patients** | 664 (100) | 483 (100) | 181 (100) |  |
| **Patient characteristics** |  |  |  |  |
| **Sex** |  |  |  | 0.000 |
| - Male | 132 (19.9) | 39 (8.1) | 93 (51.4) |  |
| - Female | 532 (80.1) | 444 (91.9) | 88 (48.6) |  |
| **Residence** |  |  |  | 0.001 |
| - Urban | 589 (88.7) | 441 (91.3) | 148 (81.8) |  |
| - Rural | 75 (11.3) | 42 (8.7) | 33 (18.2) |  |
| **Ward/Unit** |  |  |  | 0.000 |
| - Postoperative care ward /Dafra | 85 (12.8) | 84 (17.4) | 1 (0.5) |  |
| - postoperative care ward /Do | 108 (16.3) | 108 (22.4) | 0 (0.0) |  |
| - Gyn & obst postoperative care ward /CHUSS | 232 (34.9) | 230 (47.6) | 2 (1.1) |  |
| - Neonatal ward /CHUSS | 166 (25.0) | 0 (0.0) | 166 (91.7) |  |
| - ICU/CHUSS | 73 (11.0) | 61 (12.6) | 12 (6.6) |  |
| **HAI** |  |  |  | 0.000 |
| - No | 498(75.0) | 391 (81.0) | 107(59.1) |  |
| - Yes | 166(25.0) | 92 (19.0) | 74(40.9) |  |
| Length of hospital stay (days), mean, (sd) | 7.8 (7.9) | 8.3(8.2) | 6.5(6.9) | 0.011 |
| **Outcome** |  |  |  | 0.000 |
| Recoveries | 569 (85.7) | 439 (90.9) | 130 (71.8) |  |
| Death | 62 (9.3) | 16 (3.3) | 46 (25.4) |  |
| Loss to follow up | 27 (4.1) | 26 (5.4) | 1 (0.6) |  |
| Discharge against medical advice | 6 (0.9) | 2 (0.4) | 4 (2.2) |  |

Gyn & obst = Gynecology & obstetric; ICU= Intensive care unit
