# Supplementary material for: Risk and outcomes of healthcare-associated infections in three hospitals in Bobo Dioulasso, Burkina Faso, 2022: A longitudinal study
Source: PLoS One. 2025 Feb 14;20(2):e0307346. doi: 10.1371/journal.pone.0307346 (PMC11828398; doi:10.1371/journal.pone.0307346)
Supplement: Attachment — Submitted filename: Reviewers comments.docx [file pone.0307346.s005.docx]

**General comments**

Thank you so much for the opportunity to review this manuscript. It presents an interesting and important study on healthcare-associated infections (HAIs) in three hospitals in Burkina Faso, a region in West Africa that faces significant healthcare challenges, including HAIs. While the study addresses a critical need for data from resource-constrained settings, several areas require substantial revisions to improve the quality and clarity of the research presentation.

**Major comments**

1. **Language and grammar**
i. It would be beneficial to have the entire manuscript reviewed by a native English speaker or a professional editor, as there are numerous grammatical errors, spelling mistakes, inconsistent use of abbreviations and unnecessary use of bullet points (e.g., "May 1th" should be "May 1st") that distract the reader.
2. **Abstract**
i. In the "Results" section, clarify the statistical significance (e.g., p-values) of the associations mentioned.
3. **Materials and Methods**
i. Study design: Consider changing the "Study design and location" subtitle to simply "Study design" and ‘‘setting’’. Clearly outline the longitudinal, multicentre nature of the study upfront.
ii. Sampling: I suggest that the authors should include a detailed description of the sampling methods. Currently, the focus is on the wards surveyed without explaining the rationale for choosing these wards – this need to be addressed
iii. Use "exclusion criteria" instead of "non-inclusion criteria." Additionally, avoid using bullet points when describing inclusion and exclusion criteria, as this can distract the reader. These sections should be revised.
iv. Surveillance and data collection: Define the operational definitions from the French Technical Committee more clearly. What kind of modifications were made to the operational definitions? This need to be explained.
v. Provide more clarity on the method used for identifying cases and how the study ensured the reliability of data collection.
vi. Data analysis: Specify the software versions used for statistical analysis. Clarify the process by which the significance threshold was set at p < 0.05.
vii. HAI classification: The manuscript mentions targeting specific infections like SSIs and neonatal infections. However, there is a need to provide more detail about how these infections were classified and confirmed.
viii. Outcomes: The discussion around HAI outcomes, such as length of stay, needs to address the potential biases and limitations in data collection and interpretation.
4. **Results**
i. Presentation: Replace terms like "third of the sample" with specific numbers and percentages to ensure clarity.
ii. Analysis of subgroups: Provide a more detailed breakdown of HAI data by age group, sex, and hospital ward. Consider reorganising the data to separately present outcomes for children and adults.
iii. Incidence rates: The text should clarify whether the reported incidence density rates are crude or adjusted. Additionally, describe how these rates compare to those found in other regional studies to add context.

5.	**Discussion**

i. The discussion should be rewritten using a standard scientific format, including a comparison with previous research and an exploration of potential reasons for the differences observed in this study.
ii. Avoid repetition of results in the discussion section.
iii. The recommendations section seems overly general. It would benefit from being directly linked to the study's results to provide more actionable insights for healthcare facilities in similar settings.

6. **Ethics statement**

i. Expand on the ethics approval process, including the measures taken to protect patient confidentiality and the consent procedure for different age groups (especially neonates).

7. **Conclusions**

i. While the conclusions section appropriately summarises the key findings, it should be slightly expanded to include more specific recommendations for policy and practice based on the study’s results.

**Specific comments**

i. Page 4, Line 66: Consider revising to "…HAIs are caused by bacteria, viruses, or fungi that develop resistance..." and the statement should be supported with a reference.
ii. Page 9, Line 162-163: Provide more detail on how operational definitions were modified for this study. Modified operational definitions can be provided as an appendix.
iii. Clarify how patient residence (urban/rural) might influence HAI risk.

**Recommendations**

i. Restructure the methods section: Clearly define study design, setting, data collection, and analysis to facilitate reproducibility.
ii. Data analysis: Expand the data analysis section to include more in-depth explanations of the statistical methods used.
iii. Supplementary materials: Include more detailed information in the supplementary materials if some content (e.g., specific diagnostic criteria) makes the main text too dense.

Best wishes!
