# Supplementary material for: Risk and outcomes of healthcare-associated infections in three hospitals in Bobo Dioulasso, Burkina Faso, 2022: A longitudinal study
Source: PLoS One. 2025 Feb 14;20(2):e0307346. doi: 10.1371/journal.pone.0307346 (PMC11828398; doi:10.1371/journal.pone.0307346)
Supplement: Attachment — Submitted filename: Response to Reviewers.docx [file pone.0307346.s006.docx]

**Response to reviewers**

**Dear Editor and Reviewers,**

Thank you for agreeing to review this manuscript, and your comments and observations are pertinent. In this letter, we would like to provide you with some clarifications and responses to your questions.

**Academic editor**

The percentage of preventable HAI mentioned in the abstract and introduction as well as the references used to support this is debatable. Please delete this statement from the abstract. Please revise the statement in the introduction and considere the following aspects: A publication form Germany <https://www.sciencedirect.com/science/article/pii/> S0720337310001130?via%3Dihub suggests that only 30% of HAI are being preventable by hygiene measures while

**Response:** We have replaced the frequency previously mentioned in the abstract and in the text by the one reported by the German study, as recommended.

Line 50: Please correct "ESBL" producer.

**Response:** Correction applied

Your ethics statement should only appear in the Methods section of your manuscript. If your ethics statement is written in any section besides the Methods, please delete it from any other section.

**Response: Our ethics statement is in Methods section.**

**Reviewer #1:**

The Title: The use of parentheses in this title is inconvenient. Ligne 36 et 92, 131, 136 : Correct writing of dates (superscripts). Also review the entire text

**Response:** Parentheses were removed and dates were corrected as well.

Line 37: Define the abbreviation DO or write it as the name (Do). Harmonize writing throughout the text.

**Response:** We corrected and harmonized the writing of the name of the District Hospital using ''Do''.

Line 87: Risk factors (in the title) or etiological factors (as aim): For me, It seems that the terms can be nuanced.

**Response:** Correction was made: etiological factor was replaced by risk factor

Line 185: Sentence to be reviewed.

**Response**: Sentence revised

Lines 199 and 350 (ethical aspects): the reference number of the national ethics committee must be mentioned.

Response: ethics committee approval reference number was added

If you say: "We obtained informed consent, written and signed from parents or guardians for minors", please also specify if the consent of minors has also been obtained.

Response: We obtained written and signed informed consent from parents or guardians for newborns and minors. In addition to parental consent, we have obtained the assent of children over the age of 12.

Table 3: cOR and aOr must be defined in the legend.

**Response:** Correction made: cOR= crude odd ratio; aOR : adjusted odd ratio

Line 244 (point 3.3): Why were the proportions estimated based on 45 microorganisms instead of the 47 identified?

**Response:** This is an error, 45 microorganisms were identified. The correction was made.

Lines 243 and 306: How was the selection of HAIS for bacterial identification made (79 out of 166)?

**Response:** Not all HAI cases underwent biologic testing. We have randomly selected 79 patients’ sample for biological testing.

Enterobacteriaceae, Enterobacteria: Review use in context.

**Response:** Correction made, replace “Enterobacteria” with “Enterobacteriaceae

Lines 292 -305: A clearer explanation of the incidence found can be made as you have just made comparisons but without explaining why the difference in results observed. Is the comparison made with prevalence or incidence studies?

Moreover, for the articles with which the comparison is made, it would be preferable to put the results found instead of just quoting the articles in question (lines 292 -305 and 306-315).

**Response:** We have expanded the discussion to address your comments.

Ligne 303“The lack of infection control policies and trained personnel and the overcrowding of our hospitals may explain the high incidence of HAIs in our study” This aspect was not mentioned in the results section.

**Response:** correction made. The statement was deleted

Line 326-334 (penultimate paragraph of the discussion): You have mentioned the first and last names of the authors as references, which should not be the case. Please refer to the authors' guide for references in the body of the text.

**Response:** correction made in line with the authors’ guide for references in the body of the text

In conclusion: You conclude that the incidence is high. Are there any acceptable thresholds?

**Response**: Basically, there is no threshold. However, we believe that for quality care, the frequency of HAIs should be closer to 5%.

Why separate enterobacteria from gram-negative bacilli in Figure 2?

**Response:** Enterobacteriaceae are indeed Gram-negative, but this family of bacteria is of particular interest in antimicrobial resistance monitoring because of the rapid growth of ESBLE.

**Reviewer #2:** General comments

Thank you so much for the opportunity to review this manuscript. It presents an interesting and important study on healthcare-associated infections (HAIs) in three hospitals in Burkina Faso, a region in West Africa that faces significant healthcare challenges, including HAIs. While the study addresses a critical need for data from resource-constrained settings, several areas require substantial revisions to improve the quality and clarity of the research presentation.

Major comments

1. Language and grammar

i. It would be beneficial to have the entire manuscript reviewed by a native English speaker or a professional editor, as there are numerous grammatical errors, spelling mistakes, inconsistent use of abbreviations and unnecessary use of bullet points (e.g., "May 1th" should be "May 1st") that distract the reader.

**Response:** The manuscript has been proofread and corrections was made to make the text more digestible.

**2. Abstract**

i. In the "Results" section, clarify the statistical significance (e.g., p-values) of the associations mentioned.

**Response**: We have presented the adjusted odd ratios with their 95% confidence intervals. Normally, these are sufficient to show the strength of the association between HAI and patient characteristics. If a 95% CI does not contain 1, then the association is significant (p<5%).

**3. Materials and Methods**

i. Study design: Consider hanging the "Study design and location" subtitle to simply "Study design" and ‘‘setting’’. Clearly outline the longitudinal, multicentre nature of the study upfront.

**Response:** correction made accordingly

ii. Sampling: I suggest that the authors should include a detailed description of the sampling methods. Currently, the focus is on the wards surveyed without explaining the rationale for choosing these wards – this need to be addressed

**Response:** In this manuscript we present surveillance data, so we did not sample. We included all eligible cases and then measured the duration of surveillance.

iii. Use "exclusion criteria" instead of "non-inclusion criteria." Additionally, avoid using bullet points when describing inclusion and exclusion criteria, as this can distract the reader. These sections should be revised.

**Response:** Revisions was made

iv. Surveillance and data collection: Define the operational definitions from the French Technical Committee more clearly. What kind of modifications were made to the operational definitions? This need to be explained.

**Response:** We've included the operational definitions in the supplementary materials to avoid overloading the article. Please refer to the supplementary materials

v. Provide more clarity on the method used for identifying cases and how the study ensured the reliability of data collection.

**Response**: As described in the methodology, data collection teams visited the selected wards three times a week to review patient records and registers and to talk to staff and patients.

HAI cases identified by the investigators based on operational definitions were validated by a team consisting of the principal investigator, the attending physician, and the investigator teams.

vi. Data analysis: Specify the software versions used for statistical analysis. Clarify the process by which the significance threshold was set at p < 0.05.

**Response**: correction made

vii. HAI classification: The manuscript mentions targeting specific infections like SSIs and neonatal infections. However, there is a need to provide more detail about how these infections were classified and confirmed.

**Response:** Patients were classified on the basis of clinical data that was validated by the teams which comprise the treating physician and the investigative team members. They used case definitions and, where appropriate, biological data.

viii. Outcomes: The discussion around HAI outcomes, such as length of stay, needs to address the potential biases and limitations in data collection and interpretation.

**Response:** We have presented these parameters as limitations of this study in the discussion.

**4. Results**

i. Presentation: Replace terms like "third of the sample" with specific numbers and percentages to ensure clarity.

**Response:** correction made

ii. Analysis of subgroups: Provide a more detailed breakdown of HAI data by age group, sex, and hospital ward. Consider reorganising the data to separately present outcomes for children and adults.

**Response:** The sub-group analysis is shown in Table 1. It shows the distribution of HAIs cases by sex and age and ward. We have provided a table describing the distribution of adult and pediatric patients according to HAI data as supplementary information.

iii. Incidence rates: The text should clarify whether the reported incidence density rates are crude or adjusted. Additionally, describe how these rates compare to those found in other regional studies to add context.

**Response:** these are crude incidence rates.

**5. Discussion**

i. The discussion should be rewritten using a standard scientific format, including a comparison with previous research and an exploration of potential reasons for the differences observed in this study.

**Response:** The discussion was rewritten accordingly

ii. Avoid repetition of results in the discussion section.

**Response:** Comment addressed

iii. The recommendations section seems overly general. It would benefit from being directly linked to the study's results to provide more actionable insights for healthcare facilities in similar settings.

**Response:** correction made, more specific recommendations were formulated

**6. Ethics statement**

i. Expand on the ethics approval process, including the measures taken to protect patient confidentiality and the consent procedure for different age groups (especially neonates).

**Response:** correction made

**7. Conclusions**

i. While the conclusions section appropriately summarises the key findings, it should be slightly expanded to include more specific recommendations for policy and practice based on the study’s results.

**Response:** specific recommendations were included in the conclusion

**Specific comments**

i. Page 4, Line 66: Consider revising to "…HAIs are caused by bacteria, viruses, or fungi that develop resistance..." and the statement should be supported with a reference.

**Response:** reference provided

ii. Page 9, Line 162-163: Provide more detail on how operational definitions were modified for this study. Modified operational definitions can be provided as an appendix.

**Response:** correction made

iii. Clarify how patient residence (urban/rural) might influence HAI risk.

**Response:** Residence (urban/rural) had no effect on the risk of HAI in this study. However, some studies show that rural residence is associated with a higher risk of HAI. The explanation for this association remains unclear, but some authors believe that the lack of sanitary infrastructure in rural areas and the delay in access to care, especially for pregnant women, could be the cause.

**General comments**

Thank you so much for the opportunity to review this manuscript. It presents an interesting and important study on healthcare-associated infections (HAIs) in three hospitals in Burkina Faso, a region in West Africa that faces significant healthcare challenges, including HAIs. While the study addresses a critical need for data from resource-constrained settings, several areas require substantial revisions to improve the quality and clarity of the research presentation.

**Major comments**

1. **Language and grammar**
i. It would be beneficial to have the entire manuscript reviewed by a native English speaker or a professional editor, as there are numerous grammatical errors, spelling mistakes, inconsistent use of abbreviations and unnecessary use of bullet points (e.g., "May 1th" should be "May 1st") that distract the reader.

**Response:** Language issues have been addressed by proofreading the manuscript.

2. **Abstract**
i. In the "Results" section, clarify the statistical significance (e.g., p-values) of the associations mentioned.

**Response:** The p-value, or statistical significance threshold, is the probability that the observed results are due to the hazard. Conventionally, this threshold is set at 5%.

3. **Materials and Methods**
i. Study design: Consider changing the "Study design and location" subtitle to simply "Study design" and ‘‘setting’’. Clearly outline the longitudinal, multicentre nature of the study upfront.

**Response:** correction made

ii. Sampling: I suggest that the authors should include a detailed description of the sampling methods. Currently, the focus is on the wards surveyed without explaining the rationale for choosing these wards – this need to be addressed

**Response:** In this manuscript we present surveillance data, so we did not sample. We included all eligible cases and then measured the duration of surveillance.

iii. Use "exclusion criteria" instead of "non-inclusion criteria." Additionally, avoid using bullet points when describing inclusion and exclusion criteria, as this can distract the reader. These sections should be revised.

**Response:** correction made

iv. Surveillance and data collection: Define the operational definitions from the French Technical Committee more clearly. What kind of modifications were made to the operational definitions? This need to be explained.

**Response:** We didn't want to include these definitions in the article so as not to overload it. However, we have included them as supplementary material. As a modification, we did not include the different categories of site infection (superficial, deep, organ).

v. Provide more clarity on the method used for identifying cases and how the study ensured the reliability of data collection.

**Response**: As described in the methodology, data collection teams visited the selected wards three times a week to review patient records and registers and to talk to staff and patients. Selection of HAI cases was based on a flow chart (see Supplementary materials). HAI cases were validated by the survey team and the attending physician.

vi. Data analysis: Specify the software versions used for statistical analysis. Clarify the process by which the significance threshold was set at p < 0.05.

**Response**: Correction made. stata version 13 were used for data analysis. P-value statistical significance level is the probability that the observed results are due to hazard. It is conventionally set at 5%.

vii. HAI classification: The manuscript mentions targeting specific infections like SSIs and neonatal infections. However, there is a need to provide more detail about how these infections were classified and confirmed.

**Response**: classification of HAIs is based on case definitions and was validated by investigators and treating physicians.

viii. Outcomes: The discussion around HAI outcomes, such as length of stay, needs to address the potential biases and limitations in data collection and interpretation.

**Response:** We have presented these parameters as limitations of this study in the discussion.

4. **Results**
i. Presentation: Replace terms like "third of the sample" with specific numbers and percentages to ensure clarity.

**Response**: correction made

ii. Analysis of subgroups: Provide a more detailed breakdown of HAI data by age group, sex, and hospital ward. Consider reorganising the data to separately present outcomes for children and adults.

**Response:** The sub-group analysis is shown in Table 1. It shows the distribution of HAIs cases by sex and age and ward. However, a separate analysis for children and adults’ risks will take us away from the aim of the study.

iii. Incidence rates: The text should clarify whether the reported incidence density rates are crude or adjusted. Additionally, describe how these rates compare to those found in other regional studies to add context.

**Response:** these are crude incidence

5.	**Discussion**

i. The discussion should be rewritten using a standard scientific format, including a comparison with previous research and an exploration of potential reasons for the differences observed in this study.

**Response:** We have redrafted the discussion

ii. Avoid repetition of results in the discussion section.

**Response:** Repetition of results in the discussion was avoided

iii. The recommendations section seems overly general. It would benefit from being directly linked to the study's results to provide more actionable insights for healthcare facilities in similar settings.

Response: More specific recommendations were formulated

6. **Ethics statement**

i. Expand on the ethics approval process, including the measures taken to protect patient confidentiality and the consent procedure for different age groups (especially neonates).

Response: For newborns, we have obtained the consent of the parents or legal guardians.

7. **Conclusions**

i. While the conclusions section appropriately summarises the key findings, it should be slightly expanded to include more specific recommendations for policy and practice based on the study’s results.

**Response:** More specific recommendations for policy and practice based on the study’s results have been added

**Specific comments**

i. Page 4, Line 66: Consider revising to "… HAIs are caused by bacteria, viruses, or fungi that develop resistance..." and the statement should be supported with a reference.

Response: Reference provided

ii. Page 9, Line 162-163: Provide more detail on how operational definitions were modified for this study. Modified operational definitions can be provided as an appendix.

Response: correction made

iii. Clarify how patient residence (urban/rural) might influence HAI risk.

Response: residence (urban/rural) had no effect on HAI risk in this study.

**Recommendations**

i. Restructure the methods section: Clearly define study design, setting, data collection, and analysis to facilitate reproducibility.

**Response**: correction made

ii. Data analysis: Expand the data analysis section to include more in-depth explanations of the statistical methods used.

**Response**: correction made

iii. Supplementary materials: Include more detailed information in the supplementary materials if some content (e.g., specific diagnostic criteria) makes the main text too dense.

**Response**: Supplementary materials include case definition, laboratory case identification protocol, table of describing the distribution of adult and pediatric patients according to HAI data, database in order to lighten the text
