# Supplementary material for: Risk and outcomes of healthcare-associated infections in three hospitals in Bobo Dioulasso, Burkina Faso, 2022: A longitudinal study
Source: PLoS One. 2025 Feb 14;20(2):e0307346. doi: 10.1371/journal.pone.0307346 (PMC11828398; doi:10.1371/journal.pone.0307346)
Supplement: Attachment — Submitted filename: Recommendations.docx [file pone.0307346.s007.docx]

I would like to commend the authors for their efforts in addressing most of the issues raised earlier, which is highly commendable. However, I believe there is still room for improvement, particularly in refining the language to enhance clarity and flow. Below are some minor issues that could serve as a guide to address areas that have not been adequately resolved, which will improve the manuscript.

1. Writing numbers in scientific writing

In scientific writing, numbers from one to nine are typically written out in words (e.g., one, two, three) rather than as numerals (e.g., 1, 2, 3). Numbers 10 and above should be written as numerals (e.g., 10, 11, 20, etc.). Therefore, the notation such as ‘four (04)’ is redundant and should be simplified to just "four." This should be corrected throughout the manuscript.

2. Lines 124-126

The sentences ‘In 2022, the gynecology and obstetrics postoperative care ward of Sourô Sanou Teaching Hospital received 1,946 women, including 1,433 cesareans. This unit has 30 inpatient beds. The staff consisted of 16 physicians, four (04) nurses and 21 midwives.’ This can be improved as follows:

- As earlier noted, numbers between 1 and 9 should be written out in full, so "four (04) nurses" should simply be "four nurses" to avoid unnecessary distractions.
- The phrase "including 1,433 cesareans" is unclear. It might imply that all 1,946 women had cesareans, but the word "including" suggests a subset. To improve clarity, I would rephrase it to something like:

‘In 2022, the gynecology and obstetrics postoperative care ward of Sourô Sanou Teaching Hospital treated 1,946 women, 1,433 of whom had cesarean sections. The unit has 30 inpatient beds and a staff of 16 physicians, four nurses, and 21 midwives.’

Note: this is just an example

3. Lines: 152-156

The sentence ‘Information collected from these sources was supplemented by information from patients and nursing staff and recorded on individual data collection sheets which included, among others: socio demographic data (age, sex, origin, profession), factors risk related to care, initial or curative antibiotic therapy, HAI type, pathogen in question, development.’

- The phrase "factors risk related to care" is grammatically incorrect. It should be revised to something like "risk factors related to care."
- The sentence is quite long and could benefit from breaking it into two sentences to improve readability
- The phrase "included, among others" could be revised for conciseness to just "included." The reader will infer that the list is not exhaustive.
- The word "development" is vague. Specify whether it refers to patient outcomes, the progression of an infection, or something else.

4. In line 261-265

- Like other bacteria listed out the *Escherichia coli* should be italicized
- Use "spp." for species abbreviation and correct "spp1" to spp (*Pantoea spp1*)
- Use ‘spp’ instead of ‘sp’ (*Enterococcus sp*)

5. Discussion: The discussion section still contains repeated results, particularly in the first and second paragraphs, which is unnecessary. The discussion should focus on interpreting and analyzing your findings rather than restating them. While it is acceptable to highlight key results, summarizing the entire results in this section is not ideal. Please revise to ensure the discussion provides meaningful insights and context for your results.
6. Line 328: The abbreviation ‘AIS’ is not defined anywhere in the manuscript, if it refers to HAIs kindly correct it.
7. Lines 344-346: ‘Systematic empirical antibiotic therapy, whether prophylactic or curative, sometimes prior to bacterial culture, a common practice in the neonatal ward, could explain the poor performance of culture in our study.’

It is important to note that requesting a bacterial culture before surgical prophylaxis is unnecessary. Antibiotic use can generally be categorized into three main indications: empiric, targeted/definitive, and prophylactic. Prophylactic antibiotics are typically guided by the type of surgical wound (clean-contaminated or contaminated) and the likely pathogens expected during the procedure. There is ongoing debate about the necessity of prophylactic antibiotics in clean wounds, while for contaminated wounds, prophylaxis is not indicated because these wounds require treatment for an established infection. As such, obtaining a bacterial culture is not a prerequisite for surgical prophylaxis, which is specifically intended to prevent infections from anticipated pathogens rather than treat an existing one. I would suggest you revise the sentence to something like:

‘The poor performance of culture in our study could be explained by the common practice in the neonatal ward of initiating systematic empirical antibiotic therapy, prior to obtaining bacterial cultures.’

Note: This is just an example

8. Lines 358-359: ‘However, differences between men and women in terms of personal hygiene practices, of comorbidities frequency and anatomy may explain this association.’

- The phrase "in terms of personal hygiene practices, of comorbidities frequency and anatomy" is cumbersome and could be streamlined for better clarity.
- The phrase "comorbidities frequency" should be "the frequency of comorbidities" for proper grammar

I would revise the sentence as ‘However, differences between men and women in terms of personal hygiene practices, the frequency of comorbidities, and anatomy may explain this association.’

Note: This is just an example

Best of luck
