# Supplementary material for: Risk and outcomes of healthcare-associated infections in three hospitals in Bobo Dioulasso, Burkina Faso, 2022: A longitudinal study
Source: PLoS One. 2025 Feb 14;20(2):e0307346. doi: 10.1371/journal.pone.0307346 (PMC11828398; doi:10.1371/journal.pone.0307346)
Supplement: Attachment — Submitted filename: Responses to academic Editor.docx [file pone.0307346.s009.docx]

**Responses to academic Editor**

1. Please use the term “Enterobacterales” instead of “Enterobacteriaceae” in the entire manuscript.

RESPONSE: correction made

2. Please always include the country when reporting the study location including the abstract. E.g.

- […] in three hospitals in the city of Bobo Dioulasso, Burkina Faso.

- A prospective, longitudinal, multicenter study was conducted from May 1 to November 30, 2022, in two district hospitals (Do and Dafra) and the Sourô Sanou Teaching Hospital (CHUSS), Burkina Faso.

RESPONSE: correction made

3. Most importantly, please explain the fact that LOS > 2 days was identified as risk factor. What was the reference group? This does not make any sense. According to your inclusion criteria LOS > 48h is a prerequisite for inclusion. Please clarify this issue.

RESPONSE:

We agree that this section is confusing. The term "length of stay > 2 days before inclusion" refers to patients who spent more than two days in another ward in the same hospital or in another hospital before being transferred to the study ward. They become potentially eligible for our study after their transfer in one of the study wards. To be included in our study, the patient must have no signs of infection on admission and spend at least two days in one of the selected wards. Our aim was to compare patients who had spent at more than 48 hours in another ward in the same hospital or in another hospital before being admitted in the study wards (exposure group) with patients who had no such history, i.e. patients admitted directly to the study wards and those transferred to the study wards the day after admission (control group). The aim was to determine the relationship between the duration of exposure to the hospital environment and the risk of healthcare-associated infection (HAI).

We will use the term **“Previous hospital stay”** instead of “Length of hospital stays before inclusion >2 days”

4. Please use the term “risk factors” instead of “predictors” and “etiological factors” throughout the entire manuscript. Please explain in methods how the variable “Length of stay before inclusion >2 days” has been defined.

RESPONSE: correction made

5. Table 3:

- please correct “Length stays before inclusion >2 days” into “Length of stas before inclusion >2 days. Again, what was the reference here?

- Please explain the abbreviation CHUSS in the table legend.

RESPONSE: correction made
